# Does the Selected Segment Within a Two-Legged Hopping Trial Alter Leg Stiffness and Kinetic Performance Values and Their Variability?

**DOI:** 10.3390/mps8060152

**Published:** 2025-12-14

**Authors:** Ourania Tata, Analina Emmanouil, Karolina Barzouka, Konstantinos Boudolos, Elissavet Rousanoglou

**Affiliations:** Sport Biomechanics Laboratory, School of Physical Education and Sport Science, National and Kapodistrian University of Athens, 17237 Daphne, Greece; raniaptata@phed.uoa.gr (O.T.); anemmanouil@phed.uoa.gr (A.E.); kbarzouk@phed.uoa.gr (K.B.); cbountol@phed.uoa.gr (K.B.)

**Keywords:** spring-mass model, duty cycle, analysis sub-range

## Abstract

Two-legged hopping is a well-established model for assessing leg stiffness; however, in existing studies, it is unclear whether the trial segment selection affects the results. This study aimed to assess if the selected hopping segment alters the value and individual variability (%CVind) of leg stiffness and kinetic performance metrics. Elite women athletes (42, volleyball, basketball, handball) and 14 non-athletic women performed barefoot two-legged hopping (130 bpm) on a force-plate (Kistler, 9286AA, sampling at 1000 Hz). Leg stiffness was estimated from the Fz registration (resonant frequency method). Four cumulative range segments (1–10, 1–20, 1–30, and 1–40 hops) and three segments of 10-hop subranges (11–20, 21–30, and 31–40) were analyzed (repeated measures one-way Anova, *p* ≤ 0.05, SPSS v30.0). The hopping segment did not significantly alter the leg stiffness value (segment average 30.6 to 31.2 kN/m) or its %CVind (segment average ≈ 3%). The kinetic performance metrics depicted a solid foundation for the extracted leg stiffness value, with %CVind not exceeding 6.2%. The results indicate a data collection of just 15 hops, in continuance reduced to a 10 hops segment (after excluding the first five to avoid neuromuscular adaptation) as a robust reference choice.

## 1. Introduction

Two-legged hopping in place is a well-established protocol for estimating leg stiffness (a biomechanical measure of how the lower leg resists deformation), due to its repeatable, cyclic nature that imitates a spring-mass system behavior [1,2]. After the foot contacts the ground, flexion at the ankle, knee, and hip joints lowers the body’s center of mass (phase of energy absorption—spring compression). During the subsequent phase of limb extension (phase of energy release—spring recoil), the bouncing limbs generate the take-off impulse [1].

Leg stiffness influences various performance parameters [3], with the optimal level that maximizes mechanical power while maintaining injury-free performance being explored [4,5]. From the perspective of feasible yet valid leg stiffness evaluation protocols, the biomechanical importance of the hopping-in-place protocol lies in the fact that, despite its simplicity, the leg stiffness during hopping models successfully the stiffness of more complex activities such as running [1,2].

A major research concern is the impact of methodological choices in data collection and subsequent data reduction, as these may influence the accuracy and reliability of the results and, in turn, the interpretation of leg stiffness measurements. When applying a two-legged hopping protocol, one should consider the trial duration, defined either by a preset time limit [6,7,8] or by a preset number of hops [9,10,11,12], while ensuring the appropriate hopping performance [7,9,10,11,12]. In continuance, one should decide the trial segment that will be used for further analysis through a data reduction procedure, that may employ (a) the exclusion of an initial number of hops to ensure stable performance, (b) the number of critical hops that will be used for calculating the leg stiffness value, in conjunction with (c), which will be the ultimate segment along the total trial length that the critical hops will be extracted from [7,9,10,11,12,13,14,15].

Despite the widespread and well-established use of the two-legged hopping protocol for measuring leg stiffness, descriptions of data collection and reduction methods vary widely and are often not clearly justified. Although some consistency in criteria remains, variations stemming from differing research objectives lead to a lack of procedural standardization, leaving a gap that underscores the importance of establishing the optimal data reduction technique for leg stiffness values and variability.

In studies with a preset temporal limit, the trial duration variety (either with a preset temporal limit or by a preset number of hops limit) appears to associate with the protocol’s particular perspective, i.e., effect of hopping frequency [6,7,16] or loading condition [7], properties of landing surface [8,17], or landing technique [11]. Overall, the hopping trial durations may be categorized as short (<30 s) or as long (>30 up to 60 s). In studies with a preset temporal limit, a short trial duration prevails, ranging from just 4 s [7] to 10–15 s [6,8,16], for leg stiffness assessment across different hopping frequencies [6,7,16], loading conditions [7], or landing surfaces [8].

The studies where the trial duration was defined by a preset number of hops (rather than a preset temporal duration) mainly aimed the hopping frequency effect, or its interaction with gender (10 hops) Padua et al. [12], landing technique (15 hops) Lee et al. [11], and bilateral deficit or bilateral asymmetries (either during two- or single-leg hopping performance) [10,15,18]. Nevertheless, the frequency comparison itself does not forbid the choice of temporally defined trial duration. Thus, the methodological choice was somewhat arbitrary; it is not clear whether it might have impacted the results, and it hinders within-study comparison. Overall, in studies with a present number of hops, this number ranged from 15, 30, to 45 hops, indicating an overall short trial duration from 5 to 15 s; the procedures of the subsequent data reduction resulted in a shorter hop subset (segments of 5 or 10 hops) that not only varied among studies but also were not always clearly described.

For example, Padua et al. [12] citing previous studies [13,14], selected (a) the hops whose frequency was within 5% of the designated metronome frequency or the average self-selected hopping rate, and (b) hops for which the linear correlation between the vertical center of mass (COM) displacement and the vertical ground reaction force during the ground-contact phases of hopping was greater than *r* = 0.80. However, the data reduction method of Padua et al. [12] indicates that the 10-hop sub-range varied in position along the total trial length. Similarly, Lee et al. [11] used in their analysis a 15-hop segment with no clear description of its position along the trial length (in addition, the exact trial duration is most likely not explicitly stated, as the data reduction procedure involves retaining 15 consecutive hops after excluding the first and last ones.) Also, Otsuka et al. [18] reduced their 15-hop data collection to just 5 successive hops (6th to 10th hop), without explicitly stating either the 5-hop segment position along the total trial length or their selection criteria. Finally, Maloney et al. [15], like Hobara et al. [9,10,19] and Otsuka et al. [18], reduced series of 30 two-legged hops each to just 5 consecutive hops, specifically from the 6th to 10th of the total 30 hops, following the criteria of Moresi et al. [20], (that is, the ground contact time of each of the 5 hops should fall within ±5% of the average ground contact time of the 5-hop subset). Yet, given their statement that all hopping trials met the above criteria, their choice of the 5-hop subset position along the total trial length is not clearly substantiated.

When using a subset of hops rather than the total of approved hopping cycles, the segment usually comprises 3 to 10 or 15 hops (most commonly 10–15, either consecutive ones or from a dispersed selection). The conceptual convergence implies that a too short or too long hopping segment may influence the leg stiffness outcome; however, the trial segment labeling as initial, middle, and late emerges relative to the trial duration chosen by the researchers of each study, rather than being objectively and numerically defined based on previous research evidence. Variations stemming from differing research objectives may be inevitable. Yet, despite an underlying conceptual objectivity of the data reduction criteria [12,13,14,20], little is known about whether the position of the selected hopping segment along the total trial length (or the criteria themselves) may indeed affect the magnitude and the individual variability of the leg stiffness and kinetic performance metrics. When selecting a subset of human performance, the criteria imply that the selected part is “representative” of the real or optimal movement pattern [21]. This concept militates the potential functional role of variability in human movement as well as its intricate interplay with stability of performance [21,22,23,24]. The invariance of human performance, even among elite athletes, underscores the importance of methodological criteria that allow the emergence of functional and effective individual variability.

Temporal stability of kinetic performance—an index of motor control robustness—enables the reproduction of timing patterns with minimal individual variability over time. Crucially, such stability does not imply rigidity; instead, it reveals a flexible system that sustains coordination and energy efficiency under changing conditions. Preferred hopping motor frequency depicts mechanical and energetic economy [1], reinforcing and serving a self-regulating, task-sensitive system that balances motor pattern precision with adaptability [25]. Thus, the requirement to perform at a set rather than the preferred motor frequency (most often encountered in hopping studies aiming at leg stiffness assessment) may constitute a temporal biomechanical constraint that interacts with the effect of trial segments, not only affecting leg stiffness but also kinetic performance metrics. Biomechanical constraints (as the set movement frequency) do not necessarily eliminate variability; instead, they may structure it into a form of functional flexibility, enabling the timing system to adapt along the trial duration while remaining bound by the set biomechanical limit [25,26].

Thus, the purpose of this study was to assess whether the selected segment along the total of a two-legged hopping trial, that is, hopping accumulation of increasing range and consecutive hopping sub-ranges, alters leg stiffness and kinetic performance values as well as their variability.

## 2. Materials and Methods

### 2.1. Subjects

Forty-two elite female athletes—volleyball (*n* = 14; 25.3 ± 3.1 years; 74.8 ± 7.8 kg; 180.5 ± 5.9 cm), basketball (*n* = 14; 27.7 ± 6.3 years; 69.7 ± 11.8 kg; 175.9 ± 8.5 cm), and handball (*n* = 14; 21.2 ± 3.7 years; 70.1 ± 10.9 kg; 172.7 ± 3.3 cm)—and 14 healthy recreationally active women (26.7 ± 5.0 years; 65.1 ± 9.6 kg; 169.9 ± 7.3 cm) participated in the study. They all reported no lower-limb injuries in the past 12 months. The research protocol was approved by the School Bioethics Committee and adhered to the Declaration of Helsinki. Individuals had no current or recent musculoskeletal injury, joint pathology, or other medical condition that could limit the performance of repeated double-legged hops. All participants provided written informed consent before participation.

### 2.2. Experimental Procedures

Each participant was familiarized with the experimental protocol and tested in a single laboratory-based session. After recording body height using a telescopic measuring rod (Seca DE) and body weight on a calibrated force-plate (Kistler, 9286 AA, Winterthur, Switzerland), each subject performed a light-intensity 5 min warm-up (jogging, typical light stretching for the muscles of the lower extremity, and short bouts of two-legged hopping) and practiced the experimental task under supervision and guidance from the examiner. During this specific pre-test familiarization period, the investigator provided corrective feedback designed to ensure that the hopping task was performed appropriately, as described in continuance. The pre-test familiarization hopping tasks (barefoot, under metronome cueing) were performed on the force plate to familiarize participants with the 40 × 60 cm landing area. The familiarization period was followed by 2 min of rest, after which data collection was performed.

### 2.3. Double-Legged Hopping Task

**Figure 1:**
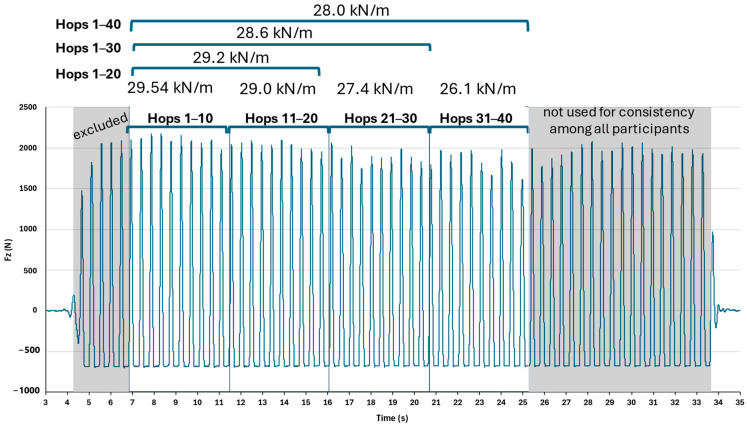
Vertical GRF (Fz in N) force-time curve during one double-legged hopping trial depicting the segment definition, for one typical athletic subject. The average of each segment is noted.

**Figure 2:**
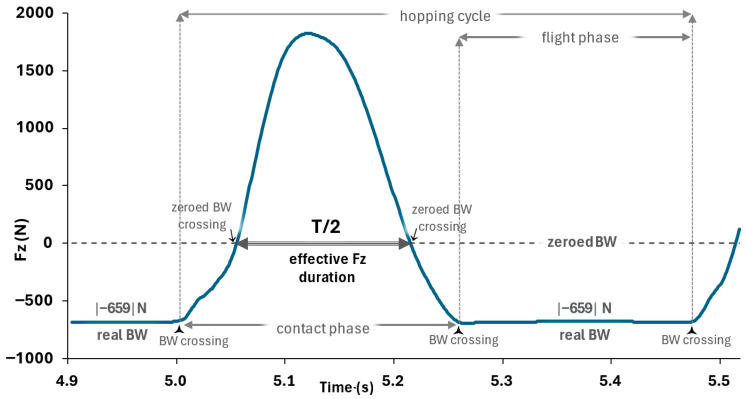
Vertical (Fz) GRF for a single hopping cycle. The definition of effective Fz duration (half the resonant period, T/2) in the formula used to calculate leg stiffness is depicted (typical athletic subject). Before data collection, with the participant standing on the force-plate, the BW recording was ‘zeroed’. Thus, the absolute value of the negative Fz resting value during the flight phase depicts the real BW value (stored in the debug window of the Kistler Bioware software used for data acquisition and analysis, Kistler Measurement, Analysis and Reporting Software v.5.5.1.0).

Each participant hopped barefoot using both legs (2 trials of two-legged hopping, 30 s each) in the middle of a 40 × 60 cm force plate (Kistler, 9286AA, sampling at 1000 Hz, Kistler Measurement, Analysis and Reporting Software v.5.5.1.0). Hands were kept at mid-waist level, feet at preferred width, and eyes directed forward. Since contact-time instructions can influence performance, stiffness, and stiffness regulation during hopping [27,28], subjects were instructed to hop naturally (minimal secondary movements in joints other than the ankle) and to land in a similar ankle position to that at take-off. To ensure a linear spring-mass behavior (sinusoidal pattern of the force-time signal) [1,2], each hopping trial was performed at 130 bpm set frequency, indicated audibly to subjects via the Tempo Perfect Metronome v.2.02a Software (available at Google Play, https://play.google.com/store/apps/details?id=com.nchsoftware.tempoperfect, assessed from the 21st of October till the 30th of November 2022). While standing and waiting for the initiation of data collection, all participants were exposed to the metronomic sound for adequate time to ensure neural entrainment for a stable motor synchronization (for a steady clear sound as the metronomic one this is expected to occur within the first 1–3 s of listening, that is first 2–10 beats) [29,30]. The set 130 bpm metronomic tempo corresponds to 2.17 Hz, the latter near or slightly above the average preferred self-selected hopping frequency for females (ref. [7], 2.07 ± 0.18 Hz; ref. [14], 2.31 ± 0.35 Hz; ref. [15], 2.8 ± 0.3 Hz; ref. [31], 2.17 ± 0.07 Hz; ref. [32], 2.05 ± 0.12 Hz). If a participant failed to perform a hopping trial adequately—e.g., landed outside the force-plate area—the trial was disregarded and repeated after a 2 min rest (only 5 participants had to repeat a trial, that is, 5 trials out of a total of 112). Given that the Fz series showed standard hopping cycle patterns (Figure 1 and Figure 2), the trial was considered valid.

### 2.4. Hopping Segment Extraction

Our research objective was to examine the effect of selection criteria. Taking into account that the instruction to synchronize with the metronome was already a performance constraint, only two strictly quantifiable criteria were applied for further analysis: (a) the validity of cycle was verified by testing the performed hopping tempo against the 130 bpm metronomic tempo (one sample *t*-test, Table A1, Appendix A) and (b) the first five hops were excluded to avoid the phase of neuromuscular adaptation [28] (a procedure applied in many previous studies [6,9,10,15,18,19,32]). From the remaining data, four cumulative hopping segments of increasing range were extracted, starting from the 1st hop and ending at the 10th, 20th, 30th, and 40th one. In addition, four consecutive 10-hop sub-ranges were extracted: hops 1st–10th, 11th–20th, 21st–30th, and 31st–40th (Figure 1). These segmentations allowed for analysis of both cumulative trial windows (increasing trial length) as well as discrete sub-ranges of consecutive hops (same trial length but at different positioning over the total trial).

### 2.5. Performed Frequency Against the Set Frequency of 130 bpm

One-sample *t*-test (with 130 bpm as the test value) was applied to examine if participants (per group, as well as per the total of participants) adhered successfully to the set metronomic tempo of 130 bpm (SPSS version 30.0, IBM statistics, significance level at a ≤0.05). As shown in Table A1 (Appendix A), across all trial segments, both per group and for the participants’ total, the performed hopping frequency did not differ significantly (*p* > 0.05) from the 130 bpm set hopping frequency. In addition, the observed hopping frequency showed non-significant group differences (Table A1—Appendix A); One-Way ANOVA for independent groups, SPSS version 30.0, IBM statistics, *p* > 0.05).

### 2.6. Variable Extraction

Hopping performance metrics. The hopping performance metrics were the peak vertical GRF (Fzpeak) expressed in BW and the durations (all expressed in s) of the hopping cycle (tcycle), contact phase (tcontact), and flight phase (tflight). Also, the duty cycle was calculated as the contact duration relative to the total hopping cycle duration and was expressed as a percentage of tcycle (%tcycle). For each kinetic performance metric, its value was calculated for every hop across the corresponding segment. The mean per segment was computed for each trial, and the average of the two trials was used in the statistical analysis.

Leg-Stiffness value. Stiffness was computed using the resonant frequency method, assuming a simple spring–mass system [31,33]. In this method, the leg compression is taken to be half the spring’s oscillation. The resonant frequency T was computed by the period indicating one half of the oscillation (T/2), that is, the duration that the net-GRF (Fi—mg) is (upwards) positive (Figure 2). In continuation, leg stiffness (k) was computed (Equation (1)) and expressed in kN/m.

(1) $$k=m{\;\times \;\omega }^{2}$$

where *ω* = 2π/Τ.

To allow a robust resonant period extraction (otherwise termed as effective vertical GRF duration), the body weight (BW) recording while the participant stood on the force-plate before each trial was ‘zeroed’ (Figure 2) aiming to apply the zerocrossing Matlab mathematical procedure (Matlab 2024a, Mathworks) for detecting the two time points defining T/2 (Figure 2—zeroed BW crossings). The BW ‘zeroing’ refers to a numerical subtraction within the data acquisition software (the BW value is subtracted from the entire Fz curve and stored in the debug window of the data acquisition software). Thus, the resting Fz value during the flight phase takes negative values equal to one BW (Figure 2). In this procedure, as depicted in Figure 1 and Figure 2, when the force plate is not loaded, the value is minus 1 BW, due to the ‘zeroing‘ procedure. However, before inserting the Fz peak values into the statistical analysis, the participant’s personal BW value stored in the software was added to each Fz peak value. To identify the contact and flight phases, the zero-crossing Matlab mathematical procedure (Matlab 2024a, Mathworks) was also applied, with BW instead of zero set as the criterion value for detection of the critical time points (Figure 2—BW crossings). Each procedure was visually inspected using plots, in which indices identified the locations of the critical time points. For all metrics, the average across the two hopping trials was used as the value for the statistical analysis.

Individual variability. In each hopping segment, for the leg-stiffness as well as the kinetic performance metrics (Fzpeak, tcontact, tflight, tcycle, and duty cycle), the relative individual variability was calculated by the coefficient of variation, that is, the ratio of the segment standard deviation (SD) to the segment average per participant, multiplied by 100 and expressed as a percentage (%CVind). For all metrics, the two trials’ mean %CVind per participant comprised the value inserted into the statistical analysis.

### 2.7. Statistical Analysis

One-way repeated measures ANOVA was applied for testing the segment effect, followed by post hoc pairwise comparisons (Bonferroni correction) in the presence of a significant segment effect. In case that Mauchly’s test indicated violation of sphericity, the Greenhouse correction was used to determine the significance of the segment effect. The Cohen’s d effect size was also computed, and the Cohen’s [34] threshold values were used for its interpretation (0.20 = small, 0.50 = medium, 0.80 = large). SPSS version 30.0 (IBM Statistics) was used for all statistical procedures with the significance level set at <0.05.

## 3. Results

Table A2 and Table A3 (Appendix A) provide all numerical details about the mean and standard deviation of the magnitude and the %CVind, respectively, as well as all indices yielded from statistical analysis concerning the group x segment interaction and the segment effect for the total of participants.

### 3.1. Group X Segment Interaction

Results are presented for the total sample of 56 participants (42 athletic and 14 non-athletic young women) because of no significant Group X Segment interaction either for the magnitude of variables (*p* > 0.05) (Table A2—Appendix A) or for their variability (*p* > 0.05) (Table A3—Appendix A).

### 3.2. Segment Effect

**Figure 3:**
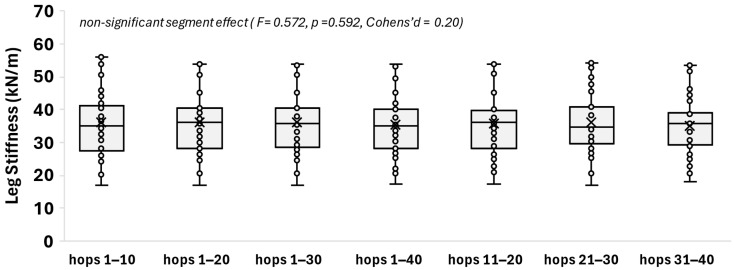
Boxplots of leg stiffness in the selected hopping segments. The box indicates the interquartile range (IQR) of the values (IQR: 50% of the values lie within 0.6745 standard deviations). Each whisker extends to the furthest data point that is within 1.5 times the IQR. The horizontal line and the x symbol in the box indicate the median and the mean, respectively, and the filled circles denote individual values (no outliers were evidenced). The segment effect statistics (F, *p* value, and Cohen’s d effect size) indicate non-statistical significance (*p* ≤ 0.05).

Figure 3 illustrates the leg stiffness expressed in kN/m across all hopping segments for the total of the 56 participants. Leg stiffness did not differ significantly (F = 0.572, *p* value = 0.592, and Cohen’s d = 0.20) among the trial segments, with average segment values ranging from about 30.6 to 31.2 kN/m.

**Figure 4:**
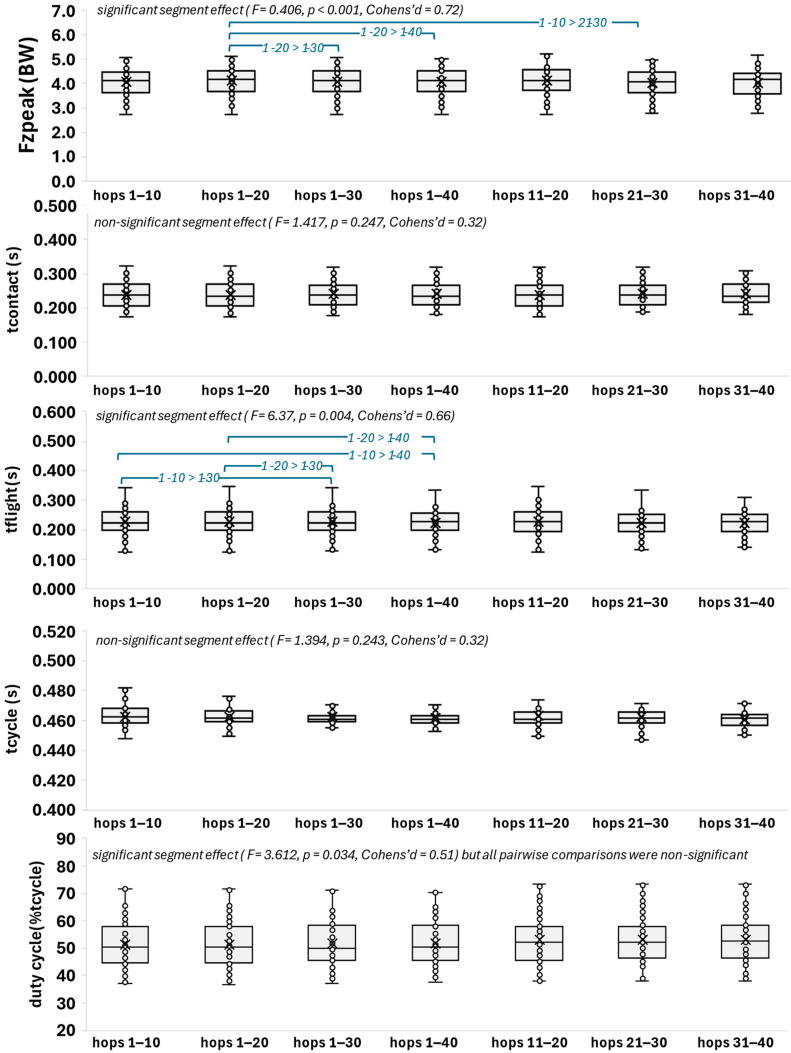
Boxplots of the two-legged kinetic performance metrics in the selected hopping segments. The box indicates the interquartile range (IQR) of the values (IQR: 50% of the values lie within 0.6745 standard deviations). Each whisker extends to the furthest data point that is within 1.5 times the IQR. The horizontal line and the x symbol in the box indicate the median and the mean, respectively, and the filled circles denote individual values (no outliers were evidenced). The segment effect statistics (F, *p* value, and Cohen’s d effect size) indicate non-statistical significance (*p* ≤ 0.05).

The trial segment effect on the kinetic performance metrics (Fz-Peak, tcontact, tflight, tcycle, and duty cycle) is depicted in Figure 4. Only Fz-peak (F = 0.406, *p* < 0.001, Cohen’s d = 0.72) and tflight (F = 6.37, *p* = 0.004, and Cohen’s d = 0.66) yielded a significant segment effect, with significant segment pairwise differences (Figure 4).

### 3.3. Trial Segment Effect on Individual Variability (%CVind)

**Figure 5:**
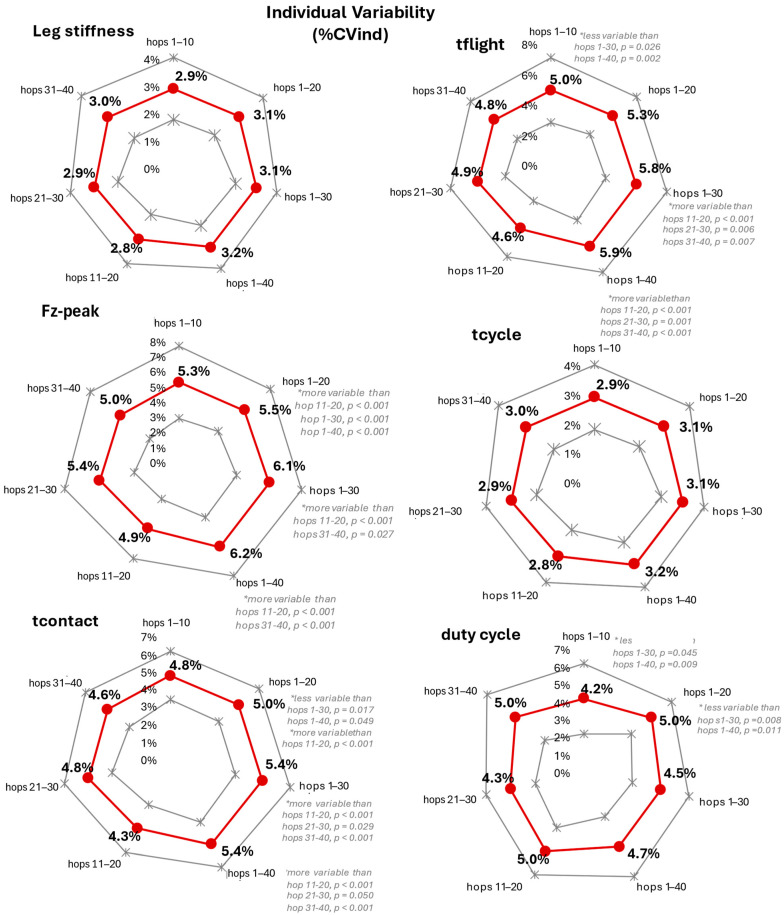
Mean (±1 SD) (red and grey indices, respectively) of individual variability (%CV) in all selected hopping segments, for leg stiffness and the kinetic performance metrics (Fz-peak, tcontact, tflight, tcycle, and duty cycle). The main effect statistics (F, *p* value, and Cohen’s d effect size) as well as the significant pairwise segment comparisons in the presence of a significant segment effect are noted. * significant difference at *p* ≤ 0.05.

The leg stiffness %CVind ranged from about 7.1% to 8.6% among the trial segments with non-significant differences among them (F = 0.784, *p* value = 0.382, and Cohen’s d = 0.20) (Figure 5) (Table A3—Appendix A). In the kinetic performance metrics, %CVind yielded a significant main effect in Fz-Peak, tcontact, tflight, and duty cycle (*p* < 0.05), with significant pairwise differences only in tcontact and tflight (Figure 5).

## 4. Discussion

The purpose of this study was to assess whether the selected segment during a two-legged hopping trial alters leg stiffness and kinetic performance values, as well as their variability. This research question was tested on a sample of elite-level athletic women (volleyball, basketball, and handball) and a cohort of non-athletic women with moderate physical activity (recreationally active, undertaking ≥ 2.5 h of physical activity per week). The results and discussion focus on the total number of participants because athletic experience (comparison between athletic and non-athletic females or among athletic groups) did not interact with the segment effect.

The leg stiffness value did not differ significantly among the trial segments, ranging from about 30.6 to 31.2 kN/m. The trial segment effect on the kinetic performance metrics was significant for Fzpeak. This finding most possibly highlights the peaking of vertical force as a key factor for motor control modulation [35]. Taken in conjunction with the significant tflight differences, the vertical force during landing may account for variations in landing velocity resulting from greater or smaller flight times. It must be noted that the whole sample of 56 participants demonstrated pure spring-mass model behavior, with the ground reaction force depicting a bell-shaped curve [13,33]. Thus, their lower-limb mechanics may be safely described as a linear spring [33].

The main finding of the study is that only the initial 15 hops (reduced to 10 after excluding the first 5 to avoid neuromuscular adaptation [28]) are sufficient for robust leg stiffness and kinetic performance results. This conclusion is supported by both the magnitude and the individual variability results. Leg stiffness is regulated through a combination of muscle activation, reflex responses, torsional stiffnesses of the joints, and joint geometry. Muscle activation levels, both before and during ground contact, are a key factor in short-contact stretch-shortening cycle activities, as joint stiffness is regulated by a change in centrally programmed muscle preactivation [27] and can be modulated by simultaneous activation of opposing muscles [36]. The gastrocnemius muscle is denoted as the muscle controlling hopping activity, with its early activation (≈85–100 ms before the foot makes contact) [27,37,38,39] being necessary to perform the negative work during landing for elastic energy storage in the Achilles tendon (although it may also play a role in generating knee flexion just before touch-down to reduce impact [37]). The tibialis anterior does not appear to play a major role in leg stiffness control [27] while the soleus [38] and the vastus lateralis [39] muscles are mostly accounted for by their reflexes. Leg stiffness also depends on the torsional stiffnesses of the joints and the geometry of the musculoskeletal system [8]. Adjustments to joint angles at the moment of the impact alters the geometry of the leg [40] thus the mechanical contribution of each joint to center of mass dynamics (damper, strut, spring, and motor-like function) [41,42] as well as the alignment of the ground reaction force vector [13] are also altered. A force vector that is more aligned with the joint’s axis of rotation will result in smaller joint moments and increased leg stiffness [43]. Thus, if the leg is more extended at the instant of touchdown, the ground reaction force vector will be more closely aligned with the joints, resulting in a decrease in joint movement and a simultaneous increase in leg stiffness. The mechanisms regulating leg stiffness were not examined in the present study; however, the results allow the inference that they were not impacted by the trial segment used to calculate the leg stiffness value.

The role and potential impact of data-reduction methods used to evaluate measures of lower limb stiffness have been previously stressed by [20], who tested during repeated maximal-effort jumping tasks in young female athletes from a variety of sport backgrounds. In hopping studies, the overall conceptual convergence implies that a too short or a too long hopping segment may influence the leg stiffness outcome; however, even for trials of the same duration, the segment labeling as initial, middle and late emerges relative to the trial duration chosen by the researchers of each study rather than from objectively and numerically standardized criteria defined by specific research evidence. Despite the overall conceptual similarity, inconsistencies remain, and little is known about whether the position of the selected segment along the total trial length may indeed affect the magnitude and/or variability of leg stiffness and kinetic performance metrics.

Overall, hopping studies use either a preset trial duration or a preset number of hops. In those with a preset trial temporal limit, trials may be categorized as short (<30 s) or as long (>30 up to 60 s). A brief trial duration appears to prevail, ranging from just 4 s [7] to 10–15 s [6,8,16] for leg stiffness assessment across different hopping frequencies [6,7,16], loading conditions [7], and landing surfaces [8]. The studies where the trial duration was defined by a preset number of hops (rather than a preset temporal duration) mainly aimed the hopping frequency effect, or its interaction, with gender Padua et al. [12] (10 hops), landing technique Lee et al. [11] (15 hops), bilateral deficit and bilateral asymmetries (employing either two- or single-leg hopping performance). In studies with a present number of hops, this number ranged from 15 to 30 or 45, indicating an overall short trial duration from 5 to 15 s, with data reduction procedures that resulted in a shorter subset of hops (segments of 5 or 10 hops) that not only varied among studies but also were not always clearly described.

The methodological choices in the literature appear inconsistent and somewhat arbitrary (most often, they do not justify the trial type or its duration, i.e., a preset temporal limit or a preset number of hops); it is not clear whether these choices might have impacted the results, and they hinder comparisons within studies. For example, Padua et al. [12] applied their data reduction method, citing previous works [13,14], yet their choice indicates a varying position along the total trial length for the 10-hop segment used in their study. Similarly, Lee et al. [11] do not clearly describe the position of the 15 hops used in their analysis along the total trial length. Otsuka et al. [18] reduced their 15-hop data collection in just 5 consecutive hops, from the 6th to the 10th of the total 15 hops, without explicitly stating either the 5-hop segment position along the total trial length or their selection criteria. Finally, Maloney et al. [15] like Hobara et al. [9,10,19] and Otsuka et al. [18], reduced series of 30 two-legged hops to just 5 consecutive hops, that is from the 6th to 10th of the total 30 hops, following the criteria of [20], (that is, the ground contact time of each of the 5 hops was required to fall within ±5% of the average ground contact time for the five-hop sample). Yet, taking their statement that all hopping trials met the above criteria, their choice for the 5-hop segment position along the total trial length is not clearly described.

The above-described inconsistencies inspired the rationale of the present study, which provides a reference for data reduction criteria in two-legged hopping studies, by testing the magnitude and variability of leg stiffness and kinetic performance metrics across different segments of a 30 s trial. These segments were defined in the perspective of increasing cumulative range (1st–10th, 1st–20th, 1st–30th, and 1st–40th, consecutive hops) as well as of 10 hop consecutive sub-ranges (1st–10th, 11th–20th, 21st–30th, 31st–40th consecutive hops). Thus, both the effect of the temporal limit (segments of the cumulative range) and the position of the segment along the trial (segments of 10-hop consecutive sub-ranges) could be tested.

Including individual variability in the present study enhances the impact of the results. Similar concerns have been raised previously about the variability in human leg stiffness across strides during running [44]. In their study, Selvitella and Foster [44] strongly recommend avoiding the sub-selection of stride lags without first confirming that sub-selection does not produce spurious results in locomotion data. Yet, to the best of our knowledge, no previous hopping study has examined individual variability across sub-ranges of trial length. It is worth noting that—among the examined hopping metrics in the present study, leg stiffness—Fzpeak and tcycle did not demonstrate significant %CVind difference among the trial segments, with leg stiffness and tcycle exhibiting the lowest one (about 3%), whereas %CVind in Fzpeak ranged from 4.6% to 6.2%. Even in the kinetic performance metrics where significant %CVind differences were evidenced among segments (tcontact, tflight, dutycycle), %CVind remained consistently low, with no metric exceeding 6.2%. Combined with the statistically proved adherence to the set hopping tempo of 130 bpm, these results indicate that, regardless of slight %CVind differences among the trial segments, the rhythmic temporal structure of two-legged hopping remained robust across the entire trial length and participants performed precisely and repeatably [22].

Individual variability provides insight into the stability of performance—an index of motor control robustness. The overall low %CVind across all tested metrics verifies the reproducibility of timing patterns over time, that is, a stable hopping performance. Crucially, such stability does not imply rigidity; instead, it reveals a flexible system that sustains coordination and energy efficiency under changing conditions (as those evidenced in the varying flight times that eventually lead to varying landing velocities, which in turn appear to be accounted for by varying the vertical force peaking). Functionally, the consistent variability across the hopping segments indicates an optimization of movement efficiency, conserving energy, and maintaining control during repetitive cyclical actions, reflecting an inherently robust coordination state—even as absolute timing adapts to task-specific demands [45].

The low %CVind of tcycle variability was expected. This metric depicts the limit cycle that the hopping cycle should be constrained to, as instructed for movement synchronization with the metronome tempo. However, the higher variation in Fz peak depicts its role in reserving tcycle stability [35] and highlights the intricate interplay among performance metrics regulating the relationship between stability and variability [23,24]. Thus, we may infer the higher variability of peak Fz serves as a flexible motor performance [21], allowing effective correction of inherent tcycle asynchronies, in the effort to maintain the synchronization of hopping performance.

In the present study, leg stiffness was calculated from the resonant frequency method, which assumes the leg compression duration as half the spring oscillation (that is, the period that the net-GRF (Fi—mg) was (upwards) positive). The resonant frequency method also assumes that, over the entire duration of the ground contact phase, the increase in the ground reaction force is linear (or nearly linear) with respect to the center of mass displacement. This method is well accepted [14,19,31,33,46]; however, the peak of Fz and the maximum leg compression do not necessarily occur at the same time [47], leading to potential over- or underestimation of leg stiffness, depending on the method used [19,48]. Furthermore, a significant interaction appears to exist between hopping frequency and leg stiffness method, showing that increasing hopping frequency reduces the differences between methods [49].

Our participants (with no significant differences among groups) peaked at Fz at about 48.1% (±3 percentage units) of contact time. The visual inspection of the GRF time curves confirmed their single-peaked, bell-shaped form across all participants, indicating linear (or near linear) spring behavior of the lower limbs; however, we did not numerically calculate the synchronization between peak Fz and the maximum compression. The lower-limb linear-spring behavior was aimed at through our methodological choice to use hopping trials constrained to a metronomic frequency of 130 bpm (that is, near or slightly above the average self-selected hopping frequency in females [7,14,15,31,32]). This choice was based on previous studies showing that, for hopping frequencies below 2 Hz, the lower limbs stop behaving as linear springs, thereby distorting the ground reaction force profile [31] as well as that, when females hop at 2 Hz, their leg stiffness is not related to passive ankle stiffness [46], thereby restricting the influence of inter-participant potential differences in ankle passive stiffness.

Hopping at preferred tempo depicts mechanical and energetic economy [1], reinforcing and serving a self-regulating, task-sensitive system that balances motor pattern precision with adaptability [25]. However, the allowance of a preferred hopping frequency (instead of a metronomically constrained one) would induce the risk of non-linear spring mass behavior. Taking into consideration that contact time instructions can influence not only stiffness regulation but also overall hopping kinetic performance [27,28], the only instruction provided to our participants was to hop with minimal secondary movements in joints other than the ankle, and to land in a similar ankle position to that of take-off.

Set hopping frequency is a common methodological choice to ensure linear spring-mass behavior; yet it may constitute a temporal biomechanical constraint that interacts with the effect of the trial segment or reduces movement variability. However, biomechanical constraints do not necessarily eliminate variability; instead, they may structure it into a form of functional flexibility, enabling the timing system to adapt over the trial duration while remaining bounded by the set biomechanical limits [25,26]. It must be noted, however, that when using a set-hopping frequency, one should carefully test the participants’ ability to perform satisfactorily at that frequency before data collection. Maloney et al. [15] report that some of their participants failed to perform at 2.2 Hz, although this is around the universally established typical human hopping frequency (ref. [7], 2.07 ± 0.18 Hz; ref. [14], 2.31 ± 0.35 Hz; ref. [15], 2.8 ± 0.3 Hz; ref. [31], 2.17 ± 0.07 Hz; ref. [32], 2.05 ± 0.12 Hz). For our participants, this was checked not only before data collection but also afterwards, using a one-sample test to contrast their hopping tempo with the metronomic tempo.

One point of interest is the absence of significant group differences between the athletic groups and between the control and each of the athletic groups. Stiffness modulation is reliant upon the task requirements, the individual’s training status, and the athletic training background of individuals [50,51]. Also, optimizing leg stiffness is necessary to enhance athletic performance. It depends on the muscle’s ability to modulate stiffness, force production, force absorption, and the rate of force production [5]. The inherently submaximal nature of the two-legged hopping task [19,32] may explain the non-significant interaction of athletic experience with the segment effect. Thus, two-legged hopping in place may be an inadequate discriminative stimulus for the athletic groups employed in the present study (court players) because of lower loading than that encountered in competitive situations with stiffer landing patterns. Millett et al. [52] compared well-trained female endurance runners with female netball players and controls across a variety of leg-stiffness-demanding tasks (including repetitive hopping); although the controls had significantly lower leg stiffness than both athletic groups, the difference between the athletic groups was also non-significant. As may be inferred from the study of Hobara et al. [19], the discriminative ability of the hopping task itself may indeed be associated with the athletes’ training backgrounds. Hobara et al. [19] found significantly higher leg stiffness in power-trained athletes than in runners, allowing the inference that, for a significant leg stiffness difference due to athletic specificity, the difference in training interventions must be at extreme mechanical or physiological limits.

Volleyball, basketball, and handball sports share a high frequency of jump-landing activities [53,54]; however, few studies employ a direct comparison of landing kinetics/kinematics among all three sports. The prevailing landing type differs among the three sports, with mostly two-legged landing in volleyball, a mix of one- and two-legged in basketball, and mainly one-legged landing in handball. The landing load is about 7 BW in volleyball [55] (with an average of 71 jumps per player per match [56]), 4 to 8 BW in basketball [57] (≈49.8 ± 20.0 jumps per player per match, dependent playing position [58]), and at about 3 BW when landing from a jump shot and about 1.5 BW when landing from a 3-step shot in handball [59] (with ~4.9 jumps per player per match [60]). Overall, the landing load during two-legged hopping in our court players (as well as in the control group) was about 4 BW, lying at the lower landing load reported for vertical high-effort jump-landing activities (countermovement jump, drop jump) (ranging from 4 to 8 BW). Thus, despite the well-established importance of two-legged hopping as a biomechanical model for predicting stiffness in more complex or more intense activities [2,61], its inherent submaximal nature may impair its discriminative ability in athletes who are systematically subjected to higher landing loads.

The stiffness values reported in the present study should be considered within the limitations of the calculation method (resonant frequency method), which, as discussed earlier, assumes synchronous occurrence of peak Fz and leg compression, which does not always hold. Such a case may result in over- or underestimation of leg stiffness; however, the leg stiffness values in our study lie within the ranges reported in previous studies. Also, the fixed hopping frequency does not allow generalization to a preferred, self-selected frequency, as the temporal constraint may restrict natural lower-limb behavior, potentially enabling the induction of significant differences across trial segments. Furthermore, a shod rather than a barefoot hopping execution could also differentiate the segment’s effect on leg stiffness and kinetic performance metrics, as well as their variability.

Ιn conclusion, regardless of training background, the leg stiffness magnitude and individual variability were both unaffected by trial segment selection, supporting the robustness of this measure across different sub-ranges of analysis. All temporal kinetic metrics were also unaffected in magnitude by trial segment selection, with overall low individual variability (despite some between-segment differences, either for segments of increasing range or of sub-range segments), indicating a consistent rhythmic performance in precision with the set hopping frequency, thus providing a robust foundation for the leg stiffness results. The significant effect on Fz magnitude most likely reflects motor control modulation accounting for variations in landing velocity (due to differences in flight time). Thus, based on the results of the present study, employing a preset hopping frequency of 130 bpm and collecting data over just 15 hops (reduced to 10 after excluding the first 5 to avoid neuromuscular adaptation) indicates the hopping frequency as a robust choice. Provided validation of the present results across a spectrum of research objectives, data-acquisition equipment, and methods used to compute leg stiffness, their practical implications lie in establishing more feasible testing procedures (either laboratory or field) through brief data acquisition that allows direct comparison among studies.

## Data Availability

The data are not publicly available due to ethical restrictions.

## Abbreviations

The following abbreviations are used in this manuscript:

GRF	Ground Reaction Force
bmp	beats per minute
SD	Standard Deviation
CV	Coefficient of Variation

## Appendix A

**Table A1:** Mean ± SD of the hopping frequency in beats per minute (bpm) in each one of the selected trial segments that was tested against the set frequency of 130 bpm. The *p*-value is noted in parentheses with the level of significance set at ≤0.05. Additionally, the statistics from the one-way ANOVA used to examine potential group differences are reported.

Trial Segments	Mean ± SD of Hopping Frequency in bpm (*p*-Value for One-Sample *t*-Test with 130 bpm as Test Value)					One-Way ANOVA for Group Effect	
	Volley (*n* = 14)	Basket (*n* = 14)	Handball (*n* = 14)	Control (*n* = 14)	Total (*n*= 56)	F	Sig.
hops 1–10	128.5 ± 3.7 (0.161)	130.6 ± 3.0 (0.430)	129.2 ± 2.3 (0.216)	129.9 ± 3 (0.863)	129.1 ± 5.0 (0.174)	1.793	0.160
hops 1–20	129.0 ± 3.7 (0.182)	130.9 ± 3.0 (0.275)	129.0 ± 2.9 (0.221)	130 ± 2.5 (1.000)	129.2 ± 4.9 (0.232)	1.822	0.155
hops 1–30	129.0 ± 3.9 (0.211)	131.3 ± 2.7 (0.101)	129.5 ± 1.9 (0.346)	129.9 ± 2.2 (0.907)	129.5 ± 4.6 (0.400)	2.034	0.121
Hops 1–40	128.4 ± 4.1 (0.293)	131.4 ± 2.8 (0.098)	129.4 ± 1.6 (0.205)	130.0 ± 2.4 (1.000)	129.6 ± 4.4 (0.544)	1.657	0.188
hops 11–20	127.9 ± 4.7 (0.199)	131.2 ± 3.4 (0.189)	128.8 ± 2.9 (0.138)	129.9 ± 2.7 (0.848)	129.3 ± 4.8 (0.259)	1.880	0.144
hops 21–30	127.8 ± 4.4 (0.287)	132.0 ± 2.8 (0.061)	130.2 ± 1.7 (0.748)	129.1 ± 3.1 (0.314)	129.8 ± 4.4 (0.711)	2.335	0.085
hops 31–40	127.6 ± 5.1 (0.681)	131.6 ± 3.0 (0.071)	130.1 ± 2.8 (0.907)	128.9 ± 6.1 (0.502)	129.9 ± 5.3 (0.915)	0.704	0.554

**Table A2:** Mean ± SD magnitude of the leg stiffness and the kinetic performance metrics in each trial segment. The statistical indices from the statistical analysis of the Group X Segment interaction, as well as the segment effect, are also reported.

	Stiffness KN/m	Fz-Peak (BW)	Tcontact (s)	Tflight (s)	Tcycle (s)	Duty Cycle (% Tcycle)
hops 1–10	31.08 ± 8.87	4.09 ± 0.57	0.240 ± 0.037	0.228 ± 0.043	0.470 ± 0.026	51.4 ± 8.2
hops 1–20	31.11 ± 8.59	4.10 ± 0.57	0.239 ± 0.037	0.227 ± 0.043	0.470 ± 0.024	51.4 ± 8.3
hops 1–30	31.07 ± 8.60	4.08 ± 0.56	0.240 ± 0.037	0.226 ± 0.042	0.468 ± 0.023	51.6 ± 8.2
hops 1–40	30.64 ± 7.92	4.07 ± 0.55	0.240 ± 0.036	0.225 ± 0.041	0.467 ± 0.022	51.7 ± 8.0
hops 11–20	31.13 ± 8.40	4.11 ± 0.58	0.239 ± 0.037	0.227 ± 0.043	0.467 ± 0.022	51.4 ± 8.3
hops 21–30	31.00 ± 8.72	4.03 ± 0.55	0.241 ± 0.037	0.222 ± 0.040	0.465 ± 0.020	52.0 ± 7.9
hops 31–40	30.65 ± 7.69	4.03 ± 0.55	0.241 ± 0.035	0.222 ± 0.038	0.437 ± 0.192	52.1 ± 7.7
**Group X Segment** **Interaction**	F = 0.795	F = 1.177	F = 1.656	F = 0.844	F = 0.940	F = 1.553
	*p* = 0.592	*p* = 0.326	*p* = 0.142	*p* = 0.489	*p* = 0.429	*p* = 0.177
**Segment Effect across the total of participants (*n* = 56)**						
**F**	0.572	8.406	1.417	6.037	1.394	3.612
**Sig.** (Greenhouse correction for all)	0.592	<0.001 *	0.247	0.004 *	0.243	0.034 * ^(with non-significant^ ^pairwise comparisons)^
**Cohen’s d effect size** [34] 0.20 = small 0.50 = medium 0.80 = large	**0.20**	**0.78**	**0.32**	**0.66**	**0.32**	0.51
	**small**	**medium to** **large**	**small to** **medium**	**medium**	**small to** **medium**	**medium**
**Partial Eta Squared**	0.010	0.133	0.025	0.099	0.025	0.062
**Noncent. Parameter**	1.340	16.230	2.841	11.110	1.415	6.666
**Observed Power**	0.151	0.956	0.299	0.855	0.214	0.632
Pairwise Segment Comparisons	ns for all	1–20 > 1–30 >1–40 >21–30	ns for all	1–10 > 1–30 >1–40 1–20 > 1–30 >1–40	ns for all	ns for all

ns: non-significant, * significant at *p* ≤ 0.05.

**Table A3:** Mean ± SD individual variability (%CVind) of the leg stiffness and the kinetic performance metrics in each trial segment. The statistical indices from the statistical analysis of the Group X Segment interaction, as well as the segment effect, are also reported.

	KN/m (%)	Fz-Peak (%)	Tcontact (%)	Tflight (%)	Tcycle (%)	Duty Cycle (%)
hops 1–10	8.2 ± 2.0	5.4 ± 2.4	4.8 ± 1.4	5.0 ± 2.1	2.9 ± 1.1	3.8 ± 1.5
hops 1–20	8.2 ± 2.1	5.5 ± 2.3	5.0 ± 1.5	5.3 ± 2.0	3.1 ± 1.1	3.9 ± 1.3
hops 1–30	8.7 ± 2.0	6.1 ± 2.2	5.4 ± 1.6	5.8 ± 2.1	3.1 ± 0.8	4.5 ± 1.5
hops 1–40	8.6 ± 2.0	6.2 ± 2.1	5.4 ± 1.4	5.9 ± 2.0	3.2 ± 0.9	4.4 ± 1.3
hops 11–20	7.1 ± 2.5	4.7 ± 2.2	4.3 ± 1.5	4.6 ± 2.0	2.8 ± 1.0	3.3 ± 1.2
hops 21–30	8.6 ± 3.0	5.4 ± 2.4	4.8 ± 1.4	4.9 ± 1.9	2.9 ± 0.9	3.7 ± 1.4
hops 31–40	7.4 ± 2.2	5.0 ± 2.6	4.6 ± 1.6	4.8 ± 2.0	3.0 ± 1.2	3.4 ± 1.3
**Group X Segment** **Interaction**	F = 8.928	F = 2.673	F = 1.492	F = 0.639	F = 1.177	F = 0.975
	*p* < 0.001	*p* = 0.035	*p* = 0.131	*p* = 0.801	*p* = 0.279	*p* = 0.471
**Segment Effect across the total of participants (*n* = 56)**						
**F**	0.784	6.648	8.182	7.955	1.222	12.803
**Sig.** (Greenhouse correction for all)	0.382	<0.001 *	<0.001 *	<0.001 *	0.303	<0.001 *
**Cohen’s d effect size** [34] 0.20 = small 0.50 = medium 0.80 = large	**0.20**	**0.70**	**0.80**	**0.80**	**0.30**	**0.97**
	**small**	**large**	**large**	**large**	**medium to small**	**large**
**Partial Eta Squared**	0.014	0.110	0.129	0.126	0.022	0.189
**Noncent. Parameter**	0.796	19.718	32.413	30.820	3.851	47.485
**Observed Power**	0.141	0.970	0.998	0.997	0.332	1.000
	ns for all	ns for all	1–20 > 11–20 >21–30 >31–40 1–30 > 11–20 >21–30 >31–40 1–40 >11–20 >21–30 >31–40	1–10 < 1–30 <1–40 1–30 > 11–20 >21–30 >31–40 1–40 > 11–20 >21–30 >31–40	ns for all	1–10 < 1–30 <1–40 1–20 < 1–30 <1–40 1–30 > 11–20 >21–30 >31–40 1–40 > 11–20 >21–30 >31–40

ns: non-significant, * significant at *p* ≤ 0.05.
